# The potential role of dynein heavy chain genes in the pathophysiology of undescended testis

**DOI:** 10.1007/s00383-026-06506-3

**Published:** 2026-06-21

**Authors:** Sinan Kılıç, Burcu Turkgenc, Yusuf Atakan Baltrak, Gülşah Can Güler, Halis Köylü

**Affiliations:** 1 Department of Physiology, Faculty of Medicine, Üsküdar University, Istanbul, Turkey; 2 Department of Pediatric Surgery, Faculty of Medicine, Istanbul Okan University, Istanbul, Turkey; 3 Department of Medical Biology, Faculty of Medicine, Üsküdar University, Istanbul, Turkey; 4 Clinic of Pediatric Urology, Hatay Training and Research Hospital, Hatay, Turkey

**Keywords:** Undescended testis, Dynein heavy chain, Pediatric surgery, Gene expression, Testicular physiology

## Abstract

**Background:**

Failures of testicular descent may result in the condition known as an undescended testis (UDT). While mechanical and hormonal causes have been researched quite extensively, not much is known about the underlying genetics. Developmental processes dependent on ciliary function may involve outer dynein arm heavy chain genes. Its role in testicular descent has never been explored. This study attempts to investigate the potential role of Outer Dynein Arm Heavy Chain (ODNAH) genes in UDT.

**Methods:**

24 male juvenile UDT patients and 24 age-matched controls undergoing circumcision were included. Tissue samples from the preputial tissue and processus vaginalis were collected during orchiopexy and circumcision, respectively. RNA was extracted, converted to cDNA, and subjected to quantitative real-time PCR analysis for five ODNAH genes: *DNAH5*, *DNAH8*, *DNAH9*, *DNAH11*, and *DNAH17* with *GAPDH* as a reference gene. The relative expression was computed using the 2^–∆∆Ct^ method.

**Results:**

The mean age of the control group was 21.9 ± 19.4 months, while that of the UDT patient group was 24.5 ± 12.3 months. There was no significant difference in age between the groups (*p* = 0.06). *DNAH9* expression was significantly higher when comparing UDT tissues to controls (*p* = 0.0021), with a 2.52-fold increase associated with its upregulation (Log2FC = 1.33). Only small, insignificant expression changes were evident for *DNAH5*, *DNAH8*, *DNAH11*, and *DNAH17*. Most patients shared a similar expression pattern for DNAH9, suggesting that it may play a role in the pathophysiology of UDT.

**Conclusion:**

These results suggest that *DNAH9* may play a role in the molecular mechanisms underlying testicular descent. The finding of *DNAH9* as a potential marker for disturbed germ cell development in cryptorchidism may represent an adaptive response to the aberrant microenvironment of UDT. These findings need to be verified by further studies, including expanded cohorts and functional analyses, to understand possible clinical relevance.

## Introduction

One of the most common congenital anomalies encountered in pediatric urology is an undescended testis. Up to 30% of preterm infants and approximately 3% of term male newborns have UDT [1]. In the first three months of life, the majority of testes spontaneously descend into the scrotum, and by the time a child reaches the age of one, the prevalence of persistent UDT has declined to approximately 1%. These abnormalities may be unilateral or bilateral and are most commonly found on the right side [2, 3].

Several theories have been put forward to explain testicular descent [4]. Normally, during fetal development, it occurs in two stages: the inguinoscrotal and the transabdominal phases. The process requires the cooperation of anatomical features like the gubernaculum and hormones such as INSL3, hCG, and testosterone. Whereas the failure of the processus vaginalis to close may lead to an inguinal hernia or hydrocele, disruption at any level in this cascade may lead to UDT [5].

Recent studies indicate that, besides hormonal and morphological variables, genetic and molecular mechanisms also play important roles in testicular descent [6]. Dyneins are motor proteins that generate forces for sliding along microtubules, promoting the movement of sperm flagella and cilia. Defects in these proteins lead to genitourinary anomalies and primary ciliary dyskinesia [7–9]. Androgen signaling and gonadal development may be affected through mutations in outer dynein arm heavy chain genes; examples include the following: *DNAH8* and *DNAH17*, which are expressed specifically in the testis [10, 11]. Thus, although hormonal and/or physical factors cause inguinoscrotal abnormalities, involvement of cilia-related molecular pathways is also suggested. However, direct evidence that supports this association remains limited.

We hypothesized that the expression of outer dynein arm heavy chain (ODNAH) genes may be dysregulated in patients with UDT. To test this hypothesis, we analyzed the expression levels of cilia-associated ODNAH genes (*DNAH5*, *DNAH8*, *DNAH9*, *DNAH11*, and *DNAH17*). The aim of this study was to determine whether such alterations in gene expression contribute to the etiology of UDT or affect the physiology of testicular descent.

## Methods

### Ethical approval and study design

This study was approved by the Istanbul Medipol University Non-Invasive Clinical Research Ethics Committee on December 13, 2024, in accordance with the Declaration of Helsinki’s ethical guidelines (Approval No: E-10840098-202.3.02-7695). All participants’ parents or legal guardians gave their written informed consent before their enrollment in the study.

### Study cohort and sample collection

A total of 48 male pediatric participants were included in the study. Twenty-four patients had UDT, and 24 were healthy age- and sex-matched controls. Participants were aged 0 to 7 years. Tissue samples were collected during surgical procedures. Processus vaginalis samples were obtained from patients undergoing orchiopexy, and preputial tissue was collected from controls during elective circumcision. The diagnosis of UDT was confirmed by physical examination and, when necessary, by ultrasonography. Cases of ascending testis were excluded.

Exclusion criteria included emergency surgery, prior inguinal procedures, urological disorders (e.g., hypospadias, hydronephrosis, vesicoureteral reflux), systemic diseases, or any contraindication to anesthesia.

The minimum sample size of 22 subjects was calculated using G*Power 3.1.9.4, based on a correlation coefficient of 0.349 from a previous study [12]. This corresponded to a medium effect size (0.59) with α = 0.05 and power (1 − β) = 0.95, confirming that the study was adequately powered.

*Surgical Procedure*: All UDT patients underwent surgery under general anesthesia with endotracheal intubation. For the control group (circumcision), procedures were performed under general anesthesia via mask or sedation without intubation. An inguinal incision was made on the right or left side. After passing through Camper’s and Scarpa’s fasciae, the inguinal canal was exposed and opened with a 0.5 cm incision. The testis was identified, mobilized from surrounding tissues (orchidolysis), and the processus vaginalis was dissected, ligated at the level of the internal ring (high ligation), and divided. The excised portion was sent for histopathological examination. The testis was then placed into a subdartos pouch in the scrotum and fixed by approximating the dartos edges.

All tissue samples were collected during surgery: processus vaginalis during orchiopexy and preputial tissue from the control group circumcisions. Right after collection, about 30 mg of each tissue sample was placed in a 1.5 ml tube with RNA-later solution (fixRNA; BMLabosis, Turkey), following the manufacturer’s guidelines to keep the RNA intact. The tissue samples kept in this solution were archived at − 20 °C for long-term preservation.

### RNA isolation and cDNA synthesis

Total RNA was isolated from the collected tissue samples using the RNeasy Mini Kit (Qiagen, Germany), following the manufacturer’s instructions with a few changes. To create a fine, uniform solution, the tissue was homogenized using 4 mm stainless steel beads at 50 Hz using the TissueLyser LT instrument (Qiagen, Germany). Before isolation, 1% beta-mercaptoethanol (BME) was added to the RLT lysis buffer.

Agarose gel electrophoresis was used to confirm the integrity of the RNA. High-quality RNA was identified by two distinct and sharp ribosomal RNA (rRNA) bands that represented the 18 S and 28 S subunits. The 28 S band had a 28 S/18S ratio of about 2.0, which was nearly twice as strong as the 18 S subunit. Since the gel showed no sign of smearing, RNA degradation was not present.

After quality assessment, isolated RNA samples underwent the synthesis of complementary DNA (cDNA) with the High-Capacity cDNA Reverse Transcription Kit (Thermo Fisher Scientific, USA) according to the manufacturer’s instructions. The remaining isolated RNA material was kept at -80 °C for long-term preservation. cDNA samples were stored at -20 °C until further processing in qRT-PCR analyses. All steps of RNA isolation, quality assessment, and subsequent cDNA synthesis were performed on ice to prevent RNA degradation.

### Quantitative real-time polymerase chain reaction (qRT-PCR) and data analysis

qRT-PCR was used to examine differences in the expression levels of *DNAH5*, *DNAH8*, *DNAH9*, *DNAH11*, and *DNAH17* in all patient and control groups. The endogenous reference (housekeeping) gene was the glyceraldehyde 3-phosphate dehydrogenase gene (GAPDH). Amplification was performed in 96-well plates using the LightCycler^®^ 480 Real-Time PCR System (Roche, Switzerland). QuantiNova Probe PCR Kit (Qiagen, Germany) was used for the amplification of the six genes (five target genes and one reference gene) of each cDNA sample, according to the recommendations of the manufacturer. QuantiNova LNA Probe PCR Assay primer–probe sets were used to evaluate the expression levels of the target and reference genes. The amplicons were between 97 and 147 bp in length. Each primer-probe set was chosen based on its RefSeq transcript, Ensembl transcript ID, and chromosomal location, respectively, corresponding to the respective target regions. The corresponding GeneGlobe identifiers are given for each assay in Table 1.

**Table 1:** List of primer–probe sets used in this study

Gene	Ref Seq	Transcript Ensembl Id	Chromosome Location	Amplicon Size	GeneGlobe ID
*DNAH5*	NM_001369	ENST00000265104	Chr.5: 13,690,331–13,944,543	97 bp	UPFH0199900
*DNAH8*	NM_001206927	ENST00000327475	Chr.6: 38,715,341–39,030,519	147 bp	UPFH0351025
*DNAH9*	NM_001372	ENST00000262442	Chr.17: 11,598,470–11,969,748	135 bp	UPFH0546244
*DNAH11*	NM_001277115	ENST00000409508	Chr.7: 21,543,215–21,901,568	115 bp	UPFH0300721
*DNAH17*	NM_173628	ENST00000389840	Chr.17: 78,423,697–78,577,394	127 bp	UPFH0353432
*GAPDH* (reference gene)	NM_00135794; NM_002046	ENST00000229239	Chr.12: 6,534,517–6,538,371	144 bp	UPFH0007663

5 µL of QuantiNova Probe PCR Master Mix (1X), 1 µL of the particular Primer-Probe Assay, 2.5 µL of the cDNA template (~ diluted to 200 ng/µL), and 1.5 µL of RNase-free H_2_O were added to each 10 µL quantitative PCR reaction mixture to complete the final reaction volume. The quantitative PCR was conducted on a LightCycler^®^ 480 Real-Time PCR System (Roche, Switzerland) with an initial enzyme activation step at 95 °C for 2 min, followed by 48 cycles of 95 °C for 5 s (denaturation) and 53 °C for 30 s (annealing and extension), with fluorescence data collected during the 53 °C step. Fluorescent signals were measured in every cycle, which gave amplification curves as a result of cDNA amplification in real-time.

### Data analysis of real-time PCR

The FC was used for relative gene expression levels, calculated according to the 2^–∆∆Ct^ formula, as described by Livak and Schmittgen in 2001 (Livak and Schmittgen, 2001). The final FC values across biological replicates were averaged using the geometric mean (Lötsch et al., 2024), which is advised for log-normally distributed expression data, while the arithmetic mean was used to average the CT values for each sample and the delta CT (ΔCT) values of the control group. This guarantees an accurate and impartial presentation of the qRT-PCR gene expression ratios.

### Statistical analysis

The distribution of age in months and ΔCT values obtained from quantitative real-time PCR results were tested for normality and homogeneity of variance before statistical comparison. The normality of distribution was further tested by the Shapiro-Wilk test through GraphPad Prism, Version 9.0.0, GraphPad Software, La Jolla, CA, USA. For all genes, variance homogeneity of the ΔCT values between patients and controls was checked by Microsoft Excel’s F-test, Variance Two-Sample for Means. These assumption tests thus informed the choice of either the parametric unpaired Student’s t-test or the non-parametric Mann-Whitney U test for group comparison. Assuming parametric conditions, an unpaired t-test with Welch’s correction was used when the variance between the two groups was unequal. Statistical significance was defined as a two-tailed p-value of less than 0.05.

### Data visualization

GraphPad Prism (Version 9.0.0; GraphPad Software, La Jolla, CA, USA) was used for all data visualizations (scatter-dot plots and bar charts). First, normalized expression levels were calculated as ΔCT = CT target – CT GAPDH to evaluate the distribution of data and the differences between the groups under study. Scatter-dot plots showing individual ΔCT values were used to visually confirm the distribution of expression and group variances before FC analysis.

Following normalization, FC was calculated using the ∆Ct approach to determine the relative differences between the patient and control groups. FC bar graphs were created based on the average FC values of the patient group. FC data were log-transformed to Log2FC to present changes in expression within a more symmetric and understandable scale. GraphPad Prism was used to create Log2FC bar graphs with the target genes on the X-axis and Log2FC values centered at zero to indicate no change in expression. Positive numbers, when compared to the control, denote up-regulation, and negative values denote down-regulation. Figures were explained for statistical significance by using standard asterisk notation: *p* < 0.05 (*), *p* < 0.01 (**), and *p* < 0.001 (***).

## Results

A total of 24 UDT patients (mean age, 24.5 ± 12.3 months; age range, 12–48 months) and 24 age- and gender-matched controls (mean age, 21.9 ± 19.4 months; age range, 8–84 months) were included in the study. Initial assessment with the Shapiro-Wilk test demonstrated that, at *p* < 0.05, the distribution of age was significantly non-normal (*p* < 0.0001) for both the UDT patient group and the control group (*p* = 0.0014). For the subsequent analysis, the chosen Mann-Whitney U test showed that there is no statistical difference in age between the patient and control groups (*p* = 0.06). Among the patients, 14 (58.3%) had right-sided, 7 (29.2%) had left-sided, and 3 (12.5%) had bilateral undescended testes.

**Fig. 1:**
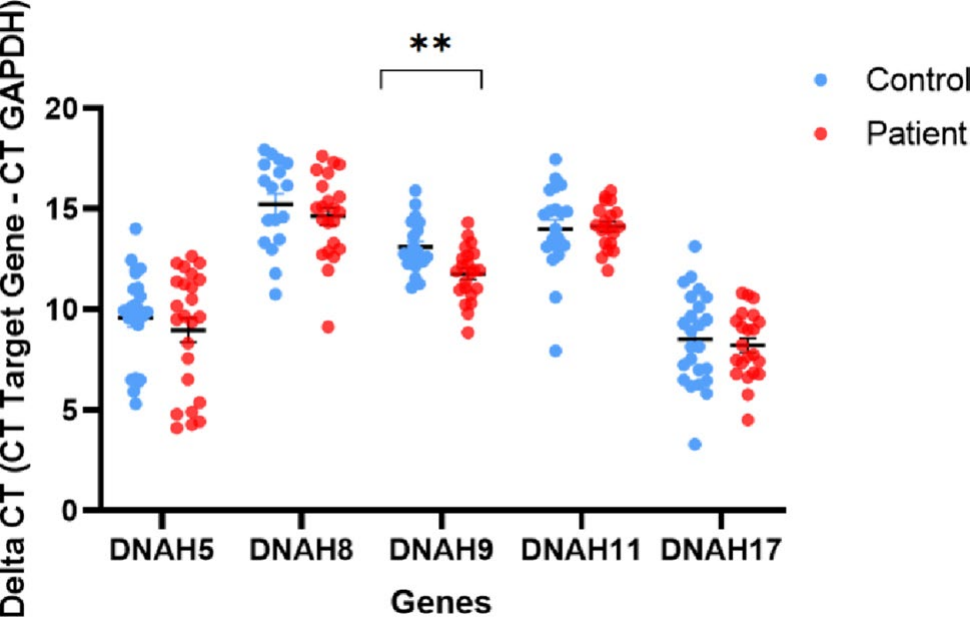
ΔCT Values of ODNAH Genes in Patient and Control Groups. This analysis presents the individual ΔCT values for the genes *DNAH5*,* DNAH8*,* DNAH9*,* DNAH11*, and *DNAH17* in both patient and control groups. Each data point represents the normalized gene expression, which is determined by subtracting the target gene’s CT value from each sample’s *GAPDH* CT value. Samples from the control group are shown by blue dots. Samples from the patient group are represented by red dots. Each group’s mean ΔCT value is represented by a center horizontal line. It is noteworthy that higher levels of gene expression are correlated with lower ΔCT values. The mean ΔCT value for the gene *DNAH9* is substantially lower in the patient group than in the control group. In the scatter plot, this difference is indicated by a double asterisk (**), which denotes statistical significance

**Fig. 2:**
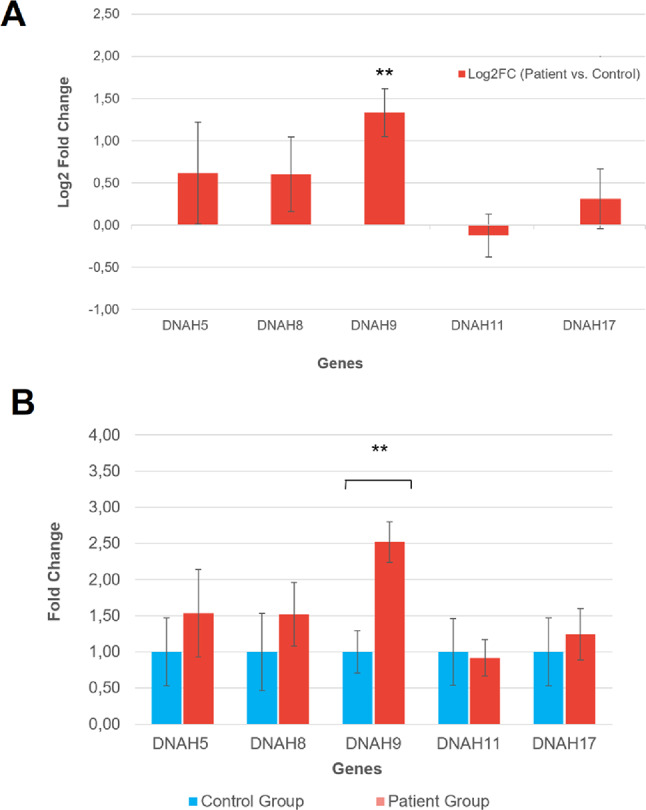
Relative Gene Expression of ODNAH Genes in Cryptorchid Patients. The control and patient groups show differential expressions of five selected ODNAH genes. SEM was inserted in error bars to indicate variability among biological replicates. The mean difference of *DNAH9* expression with an unpaired Student t-test was highly significant (*p* < 0.01) as indicated by two asterisks (**) in the bar graph **A** Log2FC expression. Bar chart showing Log2FC values for the patient relative to controls. This is asymmetric to upregulation (positive values) and downregulation (negative values). *DNAH9* had an average Log2FC of 1.33. **B** FC expression. Bar chart showing the calculated FC of the patient vs. the control groups for the ODNAH genes. The X-axis represents the analyzed genes, while the Y-axis displays the relative expression level (FC), where a value of 1.0 indicates no change in expression compared to the control group. The patient group exhibited a marked upregulation in *DNAH9* expression (FC = 2.52)

The five selected ODNAH genes (*DNAH5*,* DNAH8*,* DNAH9*,* DNAH11*, and *DNAH17*) were measured, and their differential gene expression between the patient and control groups was compared. Figures 1 and 2 show the relative expression levels of the genes, which were obtained using the FC and Log2FC methods (Table 2).

**Table 2:** Verification of Parametric Assumptions for ΔCT Values

Gene	ΔCT (Mean ± SEM)		*p*-value of the Shapiro-Wilk Test (Passed Normality T est)		*p*-value of F-test (Variance Homogeneity)	Fold Change (FC)	Log2FC (Patient vs. Control)	*p*-value	Statistical Test Used
	Control	Patient	Control	Patient					
*DNAH5*	9.61 ± 0.47	8.99 ± 0.61	*p* = 0.09 (Yes)	*P* = 0.009 (No)	Not applicable	1.53	0.62	0.68	Mann-Whitney test
*DNAH8*	15.2 ± 0.53	14.6 ± 0.44	*p* = 0.19 (Yes)	*p* = 0.30 (Yes)	*p* = 0.78 (The variances are equal)	1.52	0.60	0.39	Unpaired Student’s t-test
*DNAH9*	13.1 ± 0.29	11.8 ± 0.28	*p* = 0.46 (Yes)	*p* = 0.99 (Yes)	*p* = 0.94 (The variances are equal)	2.52	1.33	**0.0021**	Unpaired Student’s t-test
*DNAH11*	14.00 ± 0.46	14.1 ± 0.25	*p* = 0.12 (Yes)	*p* = 0.88 (Yes)	p = **0.007** (The variances are not equal)	0.92	0.12	0.82	Unpaired Student’s t-test with Welch’s correction
*DNAH17*	8.55 ± 0.47	8.23 ± 0.36	*p* = 0.91 (Yes)	*p* = 0.58 (Yes)	*p* = 0.15 (The variances are equal)	1.24	0.31	0.60	Unpaired Student’s t-test

Fold Change (FC); Log₂ Fold Change (Log2FC); delta CT (ΔCT). Statistically significant values (*p* ≤ 0.05) are shown in bold

A preliminary analysis of ΔCT values was performed for each gene in the patient and control groups. The Shapiro-Wilk test was used to determine the distribution’s normality, and the F-test was used to determine the variances’ homogeneity, which is a key assumption for certain parametric tests. Both parametric assumptions were met for *DNAH8*, *DNAH9*, and *DNAH17* (variances were equal, and *p* > 0.05 for all tests). For the final comparison, the unpaired Student’s t-test was utilized. Since the normality test revealed that the ΔCT values of the *DNAH5* gene violated the normal distribution assumption, the Mann-Whitney U test was used for comparison. Because the control group’s ΔCT values for *DNAH11* displayed unequal variances (F-test *p* = 0.007) and violated the normality assumption for both the control and patient groups, the non-parametric Mann-Whitney U test was employed for the comparison. Only the *DNAH9* gene demonstrated a significant change, according to the relative expression data, which were computed as FC and Log2FC. In the patient group, there was a statistically significant up-regulation of the *DNAH9* gene (*p* = 0.0021). The patients had a significantly lower mean ΔCT value (11.8 ± 0.28) than the controls (13.1 ± 0.29). This gives a 2.52-fold up-regulation (Log2FC = 1.33) in cryptorchid tissue. *DNAH5* and *DNAH8* both showed a slight trend of increase (FC ~ 1.5), but these differences were not statistically significant, with *p* = 0.68 and *p* = 0.39, respectively. The expression of the *DNAH17* gene was altered 1.24 times (Log2FC = 0.31). This change indicates a moderate increase; however, the difference was not statistically significant (*p* = 0.60). In the same way, no marked difference between the groups was detected for *DNAH11*, which presented a marginal variation of 0.92-fold change (Log2FC = -0.12). The results of the non-parametric Mann-Whitney U test gave further evidence for this conclusion, showing a *p* = 0.82 (Table 2).

The data were summarized using the normalized ΔCT values shown in Fig. 1 and the Log2 fold change metrics, Fig. 2. The ΔCT scatter plot, Fig. 1, displays the variation and distribution of gene expression across each sample.

Analysis of these data revealed a marked difference in the DNAH9 gene between the patient and control groups. In particular, higher expression in UDT tissue was suggested by the far lower average ΔCT value for DNAH9 in the patient group compared to the control group. By contrast, there were minimal differences in the expression levels of *DNAH5*,* DNAH8*,* DNAH11*, and *DNAH17*, as suggested by the ΔCT values for these genes that largely overlapped between the two groups (Fig. 1).

As shown in the FC bar graphs, Fig. 2B and Log2FC, Fig. 2A, further analysis measured the direction and degree of changes in gene expression. *DNAH9* was the only one to exceed the threshold for statistical significance, *p* < 0.01, with the strongest up-regulation among those genes assessed and with a mean Log2FC of 1.33, as depicted by the Log2FC chart (Fig. 2A). This is also reflected in Fig. 2B (FC plot), in which the *DNAH9* bar reaches to about 2.5 times the baseline of the control group, which is set at 1.0. Notice that the SEM bars for *DNAH9* in Fig. 2A and B are narrow, showing that these estimates are very precise and have very small deviations. On the other hand, although *DNAH5*,* DNAH8*, and *DNAH17* had positive Log2FC values, the matching bars and SEM bars in Fig. 2A significantly overlap the zero line, suggesting that there is no statistical significance in these results. Similarly, a very slight down-regulation of *DNAH11* with a Log2FC of -0.12 was shown graphically by a bar close to the zero baseline. The SEM further confirms that this slight decrease, approximately 1.09-fold, is not statistically significant.

## Discussion

The present study investigated the expression levels of ODNAH genes in the processus vaginalis of children with UDT. Our primary finding is the significant 2.52-fold upregulation of DNAH9 in UDT patients compared to healthy controls (*p* = 0.0021). To our knowledge, this is the first study to demonstrate a specific association between DNAH9 expression and the pathophysiology of UDT. While the other investigated ODNAH genes did not show statistically significant changes, the marked increase in DNAH9 suggests that dynein-related molecular pathways may play a critical role in the mechanisms governing testicular descent. These results support our hypothesis that dysregulation of motor proteins involved in ciliary function might be linked to the etiology of UDT.

Theories of testicular descent have evolved considerably since the 18th century and have predominantly emphasized the roles of anatomical structures, particularly the gubernaculum, as well as hormonal factors such as androgens and calcitonin gene-related peptide (CGRP). Although the widely accepted two-phase model, consisting of the transabdominal and inguinoscrotal stages, explains most aspects of testicular movement, the molecular mechanisms that regulate cellular motility and tissue organization during this process remain incompletely understood. Ongoing discussions concerning the inguinoscrotal phase, especially the proposed contributions of CGRP and programmed cell death within the processus vaginalis, underscore the need to identify and investigate additional molecular determinants involved in testicular descent. The theories proposed by Hutson, Tanyel, Hadziselimovic, and Husman-Levy represent the most accepted explanations for testicular descent. In this context, our findings suggest that DNAH9 may play a role in the regulatory network governing testicular descent. As a motor protein that is critical for ciliary function and microtubule-dependent intracellular transport, DNAH9 may represent a previously underrecognized molecular component of this complex developmental process [13–20]. Beyond anatomical and hormonal regulation, molecular mechanisms have recently been investigated in testicular descent [21]. Cilia-associated dyneins are motor proteins that move along microtubules and play essential roles in cellular motility, fluid transport, and tissue organization. Cilia and flagella motility is dependent on ODNAH genes. The expression of proteins involved in gonadal development and androgen signaling may be influenced by genes such as *DNAH8* and *DNAH17* [11, 22].

Among these, DNAH8 is expressed predominantly in sperm cells and not in ciliated airway epithelium. Mutations in DNAH8 have been associated with reduced sperm motility, morphological abnormalities, and even azoospermia. Whitfield et al. reported that bi-allelic mutations in DNAH17 cause isolated male infertility characterized by asthenozoospermia through a loss of dynein arm function in sperm flagella [22]. Liu et al. demonstrated that bi-allelic variants in DNAH8 lead to multiple morphological abnormalities of the sperm flagella and primary male infertility in both humans and knockout mice [11]. Yang et al. also confirmed that loss-of-function mutations in DNAH8 induce asthenoteratospermia associated with flagellar defects [23]. Tang et al. further showed that novel variants in DNAH9 can lead to nonsyndromic severe asthenozoospermia, reinforcing the crucial role of ODNAH genes in sperm motility regulation [24]. These findings indicate that abnormalities in ODNAH genes can impair sperm motility and morphology and may therefore disrupt not only fertility but also androgen-dependent genital development. Moreover, Chen et al. identified high mutation frequencies and testis-specific expression of several ODNAH genes in patients with hypospadias, supporting their possible role in the pathogenesis of undescended testis and inguinal hernia [25].

Androgen signaling, which regulates male sexual differentiation, is closely associated with gonadal development and genitourinary formation. Altered expression of ODNAH genes may therefore affect multiple developmental pathways. Inguinal hernia and undescended testis share several developmental and anatomical pathways [26]. Both conditions may result from disturbances in the normal physiology of testicular descent. If ciliary gene dysregulation interferes with this process, it might also contribute to the persistence of the processus vaginalis and the formation of an inguinal hernia. Exploring these shared mechanisms could provide a better understanding of the molecular basis of common inguinoscrotal pathologies. Dynein dysfunction that impairs microtubular motility may also disturb the function of the gubernaculum and the differentiation of androgen-dependent tissues. The molecular defects that reduce sperm motility may thus interfere with testicular descent and the closure of the processus vaginalis [27].

In the present study, expression of five selected ODNAH genes, including *DNAH5*,* DNAH8*,* DNAH9*,* DNAH11*, and *DNAH17*, was analyzed in processus vaginalis tissues after UDT surgery. Differential expression was compared between the patient and control groups. Among the analyzed genes, only *DNAH9* showed a significant increase in the patient group (*p* = 0.0021). The mean ΔCT value in patients (11.8 ± 0.28) was significantly lower than in controls (13.1 ± 0.29), indicating approximately 2.5-fold upregulation of *DNAH9* in UDT tissue (Log2FC = 1.33). *DNAH5* and *DNAH8* showed slight upward trends, but these were not statistically significant (*p* = 0.42 and *p* = 0.39). *DNAH17* increased 1.24-fold, and *DNAH11* showed a minor decrease of 0.92-fold, neither reaching statistical significance.

Unlike *DNAH9*, the absence of statistically significant variations in *DNAH5*,* DNAH8*,* DNAH11*, and *DNAH17* (*p* > 0.05) indicates that these particular ODNAH genes may not be crucial to the etiology or pathogenesis of UDT at the evaluated developmental stage [28].

This study has several limitations. Only gene expression at the tissue level was measured, and protein-level validation and/or protein localization (immunohistochemistry) of DNAH9 was not performed. The specific cell types expressing DNAH9 (e.g., gubernaculum cells, scrotal connective tissue, and inguinal canal smooth muscle cells) and the regulatory mechanisms involved remain unclear. The absence of remarkable changes in the other ODNAH genes does not preclude their possible involvement. The sensitivity of detection might be influenced by several factors, including timing, tissue type, and sample size. Due to ethical and legal constraints regarding invasive procedures in healthy children, preputial tissue was utilized as an age-matched control instead of healthy processus vaginalis. We acknowledge that the biological differences between these tissues may represent a confounding factor in gene expression analysis. Future studies should employ DNAH9-expressing cell or animal models to analyze functional roles in testicular descent. These studies should also investigate the effects of expression on scrotal positioning, seminiferous tubule development, and long-term fertility.

## Conclusion

This study provides a new perspective on the molecular basis of the undescended testis. The significant up-regulation of *DNAH9* suggests a possible malfunction in dynein-related activities, either as a secondary compensatory reaction to the hostile ectopic environment or as a basic defect causing UDT. However, further studies with larger cohorts are necessary to confirm these findings and clarify their potential clinical relevance.

## Data Availability

The datasets generated and analysed during the current study are not publicly available due to patients’ privacy, but are available from the corresponding author on reasonable request.
